# Supplementary material for: DMRforPairs: identifying Differentially Methylated Regions between unique samples using array based methylation profiles
Source: BMC Bioinformatics. 2014 May 15;15:141. doi: 10.1186/1471-2105-15-141 (PMC4046028; doi:10.1186/1471-2105-15-141)
Supplement: Additional file 2 — DMRforPairs output for the comparison of A431-MCF7 and NA17018-NA17105. Please start from the HTML files in each folder. Available via the BMC Bioinformatics website. [file 1471-2105-15-141-S2.zip › 1394847754114233_MOESM2_ESM/A431_MCF7/figures/30361.pdf]

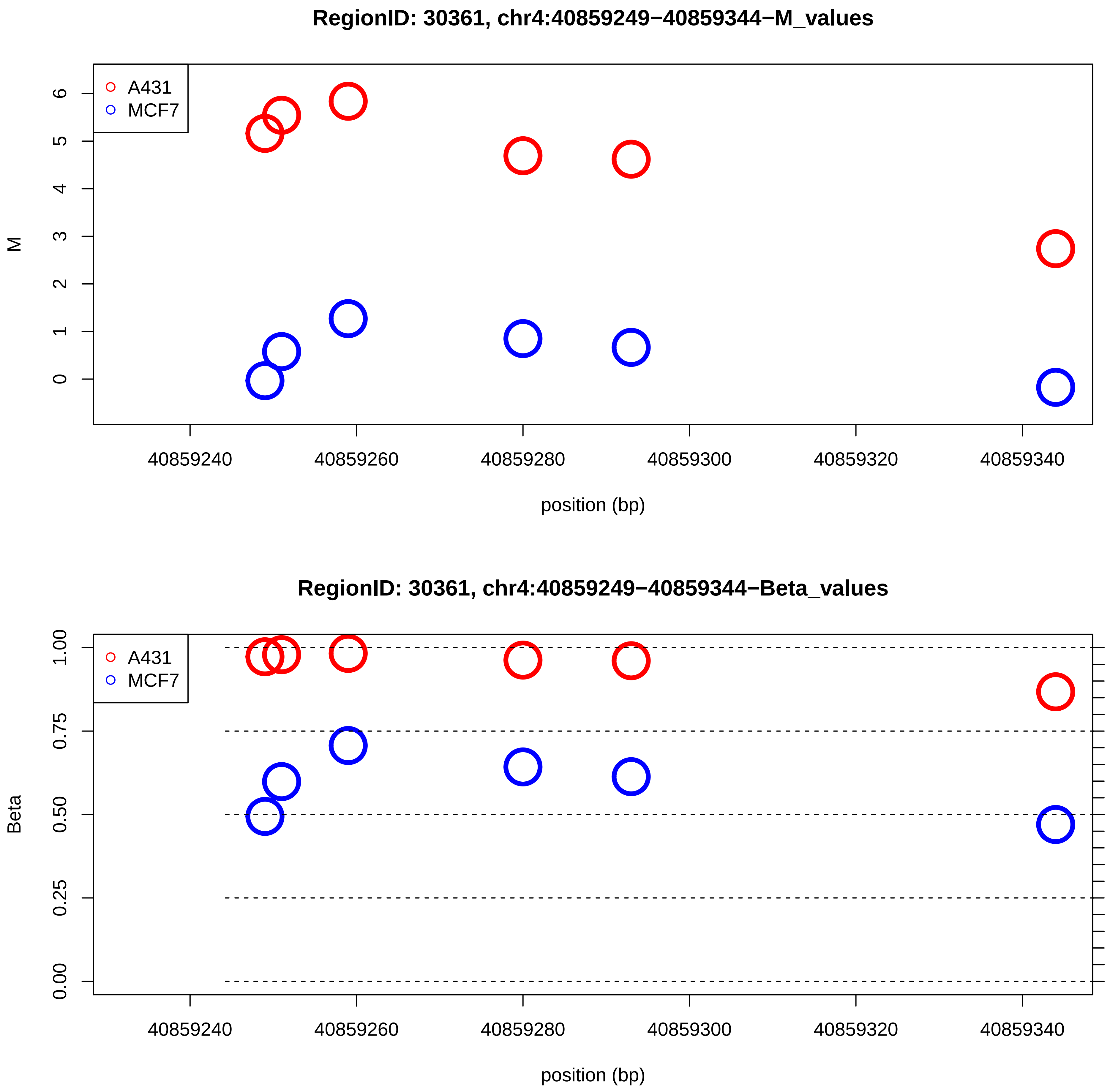
RegionID: 30361, chr4:40859249−40859344−M_values
A431
MCF7
M
0
1
2
3
4
5
6
40859240
40859260
40859280
40859300
40859320
40859340
position (bp)
RegionID: 30361, chr4:40859249−40859344−Beta_values
A431
MCF7
Beta
0.00
0.25
0.50
0.75
1.00
40859240
40859260
40859280
40859300
40859320
40859340
position (bp)

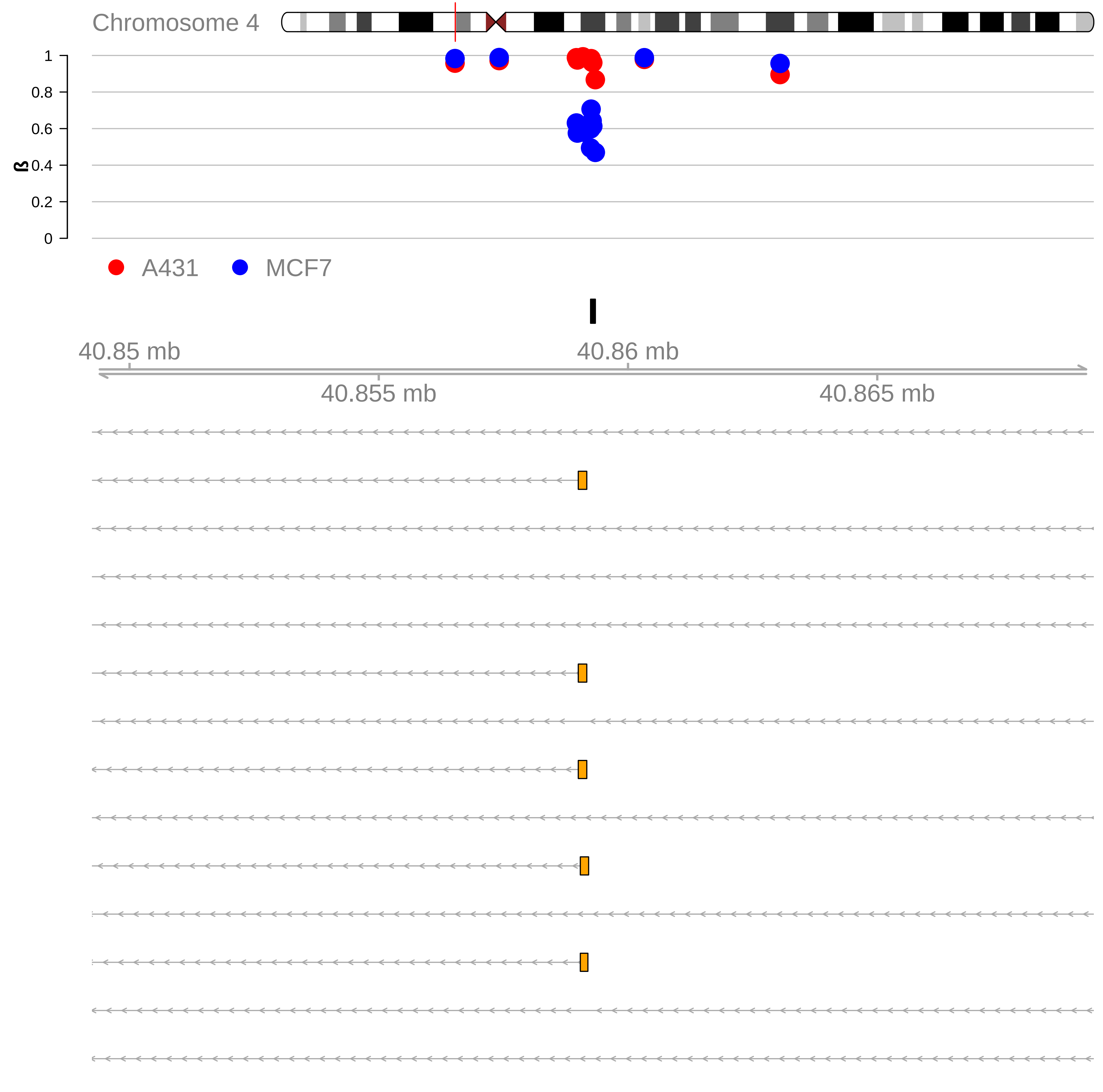

Chromosome 4
1
0.8
0.6
ß
0.4
0.2
0
A431
MCF7
40.85 mb
40.86 mb
40.855 mb
40.865 mb
